# *Chromobacterium haemolyticum*-induced bacteremia in a healthy young man

**DOI:** 10.1186/1471-2334-13-406

**Published:** 2013-09-03

**Authors:** Megumi Okada, Ryota Inokuchi, Kazuaki Shinohara, Akinori Matsumoto, Yuko Ono, Masashi Narita, Tokiya Ishida, Chiba Kazuki, Susumu Nakajima, Naoki Yahagi

**Affiliations:** 1 Department of Emergency and Critical Care Medicine, Ohta Nishinouchi Hospital, 2-5-20 Nishinouchi, 963-8558 Koriyama, Fukushima, Japan; 2 Department of Emergency and Critical Care Medicine, The University of Tokyo Hospital, 7-3-1 Hongo, 113-8655 Bunkyo-ku, Tokyo, Japan; 3 Department of Emergency and Critical Care Medical Center, Fukushima Medical University Hospital, 1 Hikariga-oka, 960-1295 Fukushima City, Japan; 4 Department of Medicine, Ohta Nishinouchi Hospital, 2-5-20 Nishinouchi, 963-8558 Koriyama, Fukushima, Japan; 5 Department of Bacteriology, Fukushima Institute of Public Health, 16-6 Mitouchi, Houkida, Fukushima city, Fukushima, Japan

**Keywords:** Chromobacterium *haemolyticum*, Chromobacterium violaceum, Sepsis, Cellulitis, Necrotizing fasciitis

## Abstract

**Background:**

The genus *Chromobacterium* consists of 7 recognized species. Among those, only *C. violaceum,* commonly found in the soil and water of tropical and subtropical regions, has been shown to cause human infection. Although human infection is rare, *C. violaceum* can cause life-threatening sepsis, with metastatic abscesses, most frequently infecting those who are young and healthy.

**Case presentation:**

We recently identified a case of severe bacteremia caused by *Chromobacterium haemolyticum* infection in a healthy young patient following trauma and exposure to river water, in Japan. The patient developed necrotizing fasciitis that was successfully treated with a fasciotomy and intravenous ciprofloxacin and gentamicin.

**Conclusions:**

*C. haemolyticum* should be considered in the differential diagnosis of skin lesions that progressively worsen after trauma involving exposure to river or lake water, even in temperate regions. Second, early blood cultures for the isolation and identification of the causative organism were important for initiating proper antimicrobial therapy.

## Background

The genus *Chromobacterium* consists of 7 recognized species: *C. violaceum*, *C. subtsugae*, *C. aquaticum*, *C. haemolyticum*, *C. pseudoviolaceum*, *C. piscinae*[1], and *C. vaccinii*[2]. Of these species, only *C. violaceum,* commonly found in the soil and water of tropical and subtropical regions, has been shown to cause human infection. Although human infection is rare, *C. violaceum* can cause life-threatening sepsis, with metastatic abscesses, most frequently infecting those who are young and healthy [3].

We describe, here, clinical evidence for another species, *C. haemolyticum*, which caused an infection in a healthy, young patient*.* This bacterium should be considered in the differential diagnosis of skin lesions that quickly worsen after trauma associated with exposure to river or lake water. This is particularly important because of the species’ resistance to antimicrobials, particularly β-lactams.

## Case presentation

A healthy, 26-year-old Japanese man was admitted to our hospital with pain in his left shoulder and leg caused by a road accident; he was hit by a car and thrown into a river. The outside air and water temperatures at the time of the injury were 20°C. He had no family history of any particular condition.

Upon admission, his temperature was 36.6°C; pulse rate, 66 beats/min; blood pressure, 143/99 mmHg; respiratory rate, 15/min; oxygen saturation, 100% (10-L reservoir mask); and Glasgow Coma Scale score, E4V5M6. A physical examination showed only bruising, not open wounds, on the left side of his face and shoulder and tenderness in his left leg. His height, weight, and body mass index were 175 cm, 67 kg, and 21.9 kg/m^2^, respectively. Laboratory tests showed mild inflammation (white blood cell [WBC] count, 13,500/μL; and C-reactive protein [CRP], 0.9 mg/L [normal range: < 2.0 mg/L]). A radiograph showed fractures of the fibula shaft and scapula.

**Figure 1:**
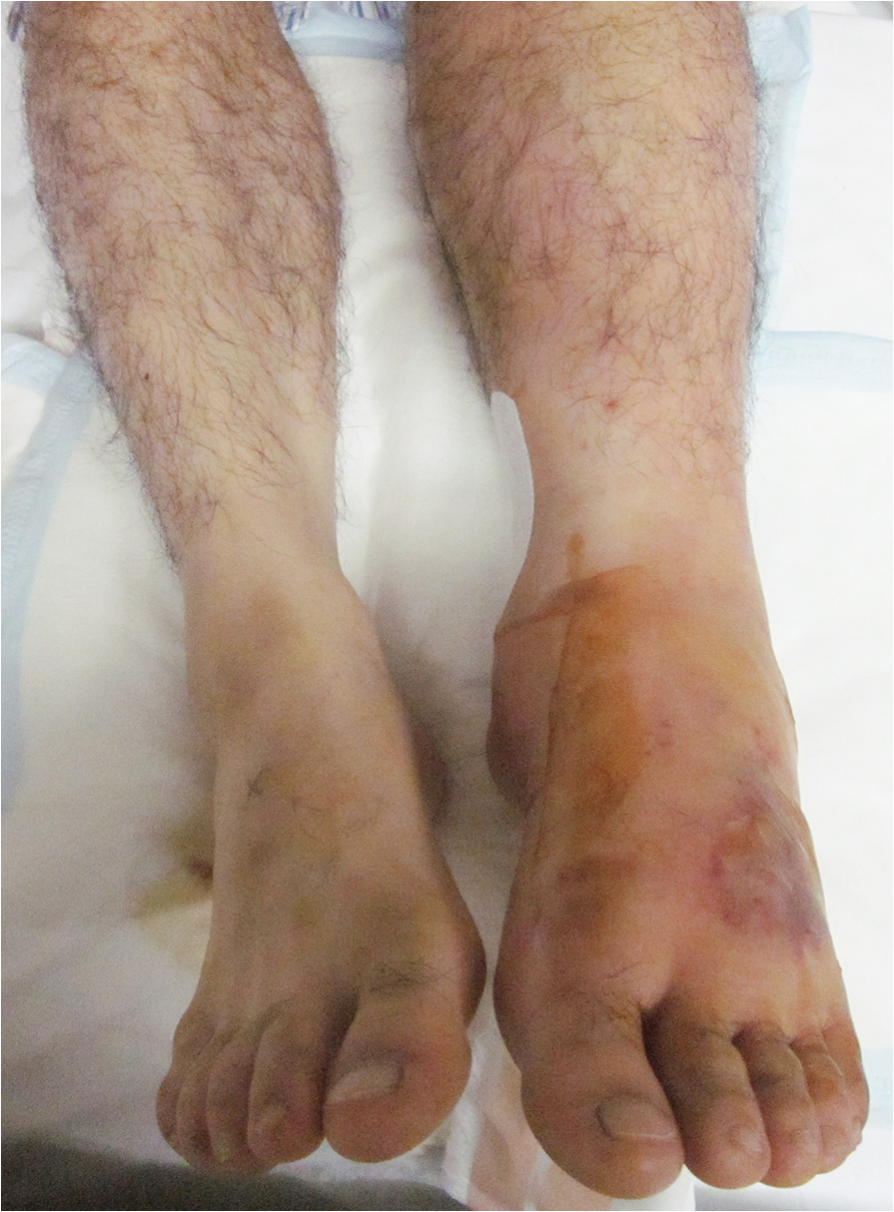
**Clinical presentation on day 6.**The patient experienced a burning sensation, tenderness, and redness in his left leg, from the upper part of his foot to his knee. Blisters are shown on the dorsum of the foot.

**Figure 2:**
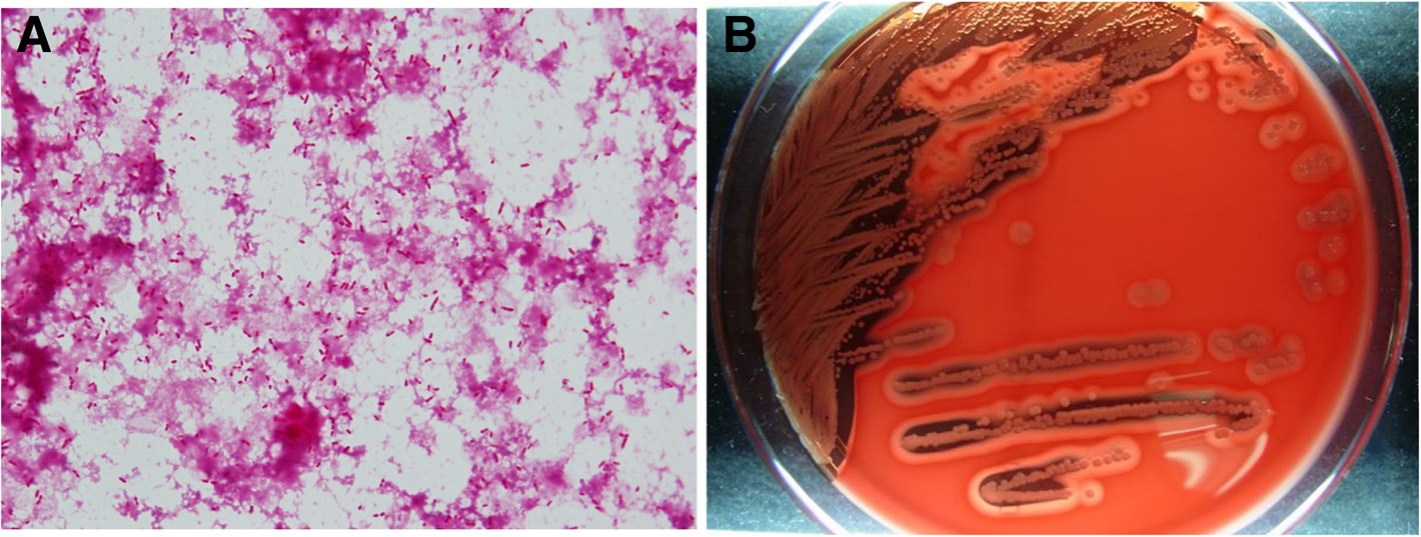
**Microbiological results. A**. A large number of short, tapered, gram-negative bacilli were present in fasciotomy. (Gram stain, ×400 original magnification). **B**. *Chromobacterium haemolyticum* colonies, showing marked hemolysis on sheep blood agar after a 24-h culture.

On post-admission day 3, the patient exhibited a temperature of 40°C, shivering, and redness and pain in his ankle. Intravenous ampicillin/sulbactam (6 g/day) and minocycline (200 mg/day) were initiated. However, on the fourth day of admission, the antimicrobial drugs were changed to ceftazidime at 4 g/day, as 2 sets of blood culture tests revealed gram-negative bacilli. Until then, the redness in his ankle had not expanded. On day 5, the erythematous, warmth and pain spread to his knee joint, and blisters were observed on the upper part of his foot (Figure 1). The patient was diagnosed with necrotizing fasciitis and a fasciotomy was conducted. The underlying muscle appeared healthy, and further debridement was avoided. The pathology of the dorsum of his left foot revealed inflamed skin and soft tissue, with necrosis consistent with necrotizing fasciitis. Laboratory tests showed an inflammatory reaction (WBC count, 13,500/μL; CRP, 196.3 mg/L), and hyperglycemia (192 mg/dL). Thus, the Laboratory Risk Indicator for Necrotizing Fasciitis (LRINEC) score [4] was 6 (intermediate risk). The smear from the fasciotomy site and blood culture tests revealed gram-negative bacilli (Figure 2A), and the infecting bacterium was oxidase-positive and produced grey colonies, with a 4.3-mm hemolytic zone on sheep blood-agar plates (Figure 2B); non-pigmented colonies were found on bromothymol-blue lactose agar plates, suggesting that *C. haemolyticum* was the causative agent. However, the Vitek GNI + card (bioMérieux Vitek, Hazelwood, MO, USA) and the API 20 NE test (bioMérieux Vitek) identified the organism as *C. violaceum*. The antibiotics were changed to intravenous ciprofloxacin (900 mg/day) and gentamicin (400 mg/day [6 mg · kg^-1^ · day^-1^]). This antimicrobial regimen was continued for 28 days. After that, gentamicin was discontinued. The trough value of gentamicin was 0.3 μg/mL (6.1 μmol/L) on days 8, 10, and 18. On day 33, the patient was discharged with a 14-day prescription for oral ciprofloxacin (1600 mg/day [25 mg · kg^-1^ · day^-1^]). Therefore, this patient was treated with gentamicin for 23 days, intravenous ciprofloxacin for 28 days, and oral ciprofloxacin for 14 days. Subsequently, the identification of the *C. haemolyticum* strain, MDA0585^T^, was confirmed, based on its 16S rRNA gene sequence. Six months after the accident, the patient was free of recurrent infection.

## Discussion

Before the recognition of *C. haemolyticum* in 2011, less than 140 proven cases of human infection with *C. violaceum* had been reported [3]. Among the few reports of infections caused by non-pigmented, β-hemolytic strains of *C. violaceum*, identification of *C. haemolyticum* strain MDA0585^T^ by 16S rRNA gene sequencing has not been previously reported. Thus, we present the first clinical evidence for *C. haemolyticum* infection.

*C. haemolyticum*, strain MDA0585^T^, is a gram-negative bacillus, and has been isolated from a clinical sample [5] and from lake water [1]. *C. violaceum* and *C. haemolyticum* are closely related, phylogenetically, making them impossible to distinguish based on results of biochemical tests (i.e., the Vitek GNI + card and the API 20 NE test)*.* However, the lack of violet pigmentation, hemolysis of sheep blood, and positive oxidase test results allow an accurate identification. Hence, as shown in the current report, these features can be used to differentiate between these 2 bacterial species in a clinical setting.

We have investigated the minimum inhibitory concentration for *C. haemolyticum.* In addition, Han *et al.* showed differences in the antibiotic susceptibility of *C. haemolyticum* and *C. violaceum* in clinical samples; hence, we compared them and the results are shown in Table 1[5]*. C. haemolyticum* was more resistant, overall, with higher minimum inhibitory concentrations for most drugs. Thus, the ability to differentiate between these 2 bacterial species is important when infection with *C. haemolyticum* is suspected because of its greater resistance to antimicrobials, especially the β-lactams [5].

**Table 1:** **Growth, biochemical reactions, and results of antibiotic susceptibility tests for *C. haemolyticum *isolates**

	**Present case**	**Xan *et al.***[5]	
Characteristic	*C. haemolyticum*	*C. haemolyticum*	*C. violaceum*
Growth on agar (37°C, 5% CO_2_, 24 h):	Non-pigmented	Non-pigmented	Purple
Sheep blood	2.3 mm, β*-*hemolysis	2 mm, β*-*hemolysis	2 mm, no hemolysis
Chocolate	2.9 mm	2 mm	2 mm
Buffered charcoal yeast extract	NA	2 mm, flat, dull	2 mm, raised, shiny
Trypticase soy	2.9 mm	1.8 mm	1 mm
MacConkey	NA	0.2 mm, pinpoint	1 mm
Hektoen enteric	NA	No growth	0.2 mm, pinpoint
Indole production (tryptophanase)	-	-	+
Glucose fermentation	NA	+ (weak)	+
Utilization of:			
Mannose	-	-	+
Mannitol	+	+	-
Citrate	+	+	-
Oxidase	+	+	-
Catalase	-	+ (weak)	+
Antimicrobial susceptibility (MIC, μg/mL)			
Amikacin	>32	16, S	3, S
Cefepime	NA	>32, R	2, S
Ceftriaxone	NA	>32, R	>32, R
Ciprofloxacin	<1	0.003, S	0.006, S
Imipenem	2	6.0, S	1.0, S
Penicillin	NA	>32, R	>32, R
Piperacillin/tazobactam	NA	>256, R	3, S
Ticarcillin/clavulanate	NA	>256, R	32, I
Trimethoprim/sulfamethoxazole	NA	0.094, S	0.094, S

*MIC* minimum inhibitory concentration, *NA* not assessed.

In the present report, the patient was found to have necrotizing fasciitis on day 6 based on his clinical presentation. In terms of antibiotic therapy for necrotizing fasciitis, currently acceptable regimens include the admin-istration of a carbapenem or β-lactam/β-lactamase inhibitor, together with clindamycin, in addition to an agent with activity against methicillin-resistant *S. aureus*[6]. In addition, the Gram stain showed the bacteria to be gram-negative. Hence, without the results of the blood culture taken on day 4, we would likely have concluded that the infection was caused by *Burkholderia* spp., *Aeromonas* spp., or *Pseudomonas* spp. [7], and would have administrated antibiotics according to the therapy for general necrotizing fasciitis. However, *C. haemolyticum* has been found to be extremely resistant to beta-lactams. Therefore, as our initial findings suggested an infection with either *C. haemolyticum* or the closely related *C. violaceum*, we provided long-term ciprofloxacin. According to a study by Aldridge *et al*., ciprofloxacin was the most active drug in combatting *C. violaceum*[8], and several studies have reported successful treatment with this therapy.

The most common symptoms of *C. violaceum* infections are fever and pain over the infected site, in association with various skin lesions [9]. To our knowledge, only 1 case of necrotizing fasciitis involving *C. violaceum* has been reported [10]. According to the pathology results, the patient in the current report had necrotizing fasciitis. Chattopadhyay *et al.* have recommended aggressive debridement for cases of *C. violaceum* infection [11].

## Conclusions

*C. haemolyticum* should be considered in the differential diagnosis of skin lesions that progressively worsen after trauma involving exposure to river or lake water, even in temperate regions. Second, early blood cultures for isolation and identification are important for initiating proper antimicrobial therapy.

### Consent

Written informed consent was obtained from the patient for publication of this case report and the accompanying images. A copy of the written consent is available for review by the Editor of this journal.

## Competing interests

The authors declare that they have no competing interests.

## Authors’ contributions

MO, KS, YO, MN, TI, and AM contributed to patient management. MN and CK performed the microbiological culturing and identification. MO and RI drafted the initial manuscript. YO, MN, TI, and AM contributed to writing the report. KS, SN, and NY critically reviewed the manuscript. All the authors have provided written consent for publication. All authors read and approved the final manuscript.

## Pre-publication history

The pre-publication history for this paper can be accessed here:

http://www.biomedcentral.com/1471-2334/13/406/prepub

## References

[B1] Lima-Bittencourt CI Costa PS Barbosa FA Chartone-Souza E Nascimento AM Characterization of a *Chromobacterium haemolyticum* population from a natural tropical lake Lett Appl Microbiol 2011 52 642 650 10.1111/j.1472-765X.2011.03052.x 21466570

[B2] Soby SD Gadagkar SR Contreras C Caruso FL *Chromobacterium vaccinii* sp. nov. isolated from native and cultivated cranberry (*Vaccinium macrocarpon* Ait.) bogs and irrigation ponds Int J Syst Evol Microbiol 2012 63 1840 1846 22984138 10.1099/ijs.0.045161-0

[B3] Yang CH Li YH *Chromobacterium violaceum* infection: a clinical review of an important but neglected infection J Chin Med Assoc 2011 74 435 441 10.1016/j.jcma.2011.08.013 22036134

[B4] Sultan HY Boyle AA Sheppard N Necrotising fasciitis BMJ 2012 345 e4274 10.1136/bmj.e4274 22822005

[B5] Han XY Han FS Segal J *Chromobacterium haemolyticum* sp. nov., a strongly haemolytic species Int J Syst Evol Microbiol 2008 58 1398 1403 10.1099/ijs.0.64681-0 18523185

[B6] Mandell G Bennett J Dolin R Mandell, Douglas, and Bennett’s Principles and Practice of Infectious Diseases, 7th Edition 2010 Philadelphia: Churchill Livingston

[B7] Campbell JI Lan NP Qui PT Dung LT Farrar JJ Baker S A successful antimicrobial regime for *Chromobacterium violaceum* induced bacteremia BMC Infect Dis 2013 13 4 10.1186/1471-2334-13-4 23286235 PMC3543835

[B8] Aldridge KE Valainis GT Sanders CV Comparison of the in vitro activity of ciprofloxacin and 24 other antimicrobial agents against clinical strains of *Chromobacterium violaceum* Diagn Microbiol Infect Dis 1988 10 31 39 10.1016/0732-8893(88)90124-1 3168426

[B9] De Siqueira IC Dias J Ruf H Ramos EA Maciel EA Rolim A Labur L Vasconcelos L Silvany C *Chromobacterium violaceum* in siblings, Brazil Emerg Infect Dis 2005 11 1443 1445 10.3201/eid1109.050278 16229777 PMC3310629

[B10] Seigel JK Stadler ME Lombrano JL Almony JS Couch ME Belhorn TH *Chromobacterium violaceum* necrotizing fasciitis: A case report and review of the literature Ear Nose Throat J 2012 91 479 483 23288793 10.1177/014556131209101108

[B11] Chattopadhyay A Kumar V Bhat N Rao P *Chromobacterium violaceum* infection: A rare but frequently fatal disease J Pediatr Surg 2002 37 108 110 10.1053/jpsu.2002.29439 11781998

